# Identification of QTLs for Salt Tolerance at the Germination and Seedling Stages in Rice

**DOI:** 10.3390/plants10030428

**Published:** 2021-02-24

**Authors:** Walid Raafat Nakhla, Wenqiang Sun, Kai Fan, Kang Yang, Chaopu Zhang, Sibin Yu

**Affiliations:** 1 National Key Laboratory of Crop Genetic Improvement, Huazhong Agricultural University, Wuhan 430070, China; walidraafat@webmail.hzau.edu.cn (W.R.N.); wqsun@webmail.hzau.edu.cn (W.S.); Fankai_1995@126.com (K.F.); yangkfor@163.com (K.Y.); zchaopu@163.com (C.Z.); 2 College of Plant Science and Technology, Huazhong Agricultural University, Wuhan 430070, China

**Keywords:** Africa cultivated rice, salt tolerance, seedling establishment, quantitative trait loci (QTLs), backcross inbred lines (BIL)

## Abstract

Rice is highly sensitive to salinity stress during the seedling establishment phase. Salt stress is widely occurring in cultivated areas and severely affects seed germination ability and seedling establishment, which may result in a complete crop failure. The objective of the present study is to identify quantitative trait loci (QTLs) related to salt tolerance of the germination and seedling stages in a rice backcross inbred line (BIL) population that was derived from a backcross of an Africa rice ACC9 as donor and *indica* cultivar Zhenshan97 (ZS97) as the recurrent parent. Under salt stress, ACC9 exhibited a higher germination percentage, but more repressed seedling growth than ZS97. Using the BIL population, 23 loci for germination parameters were detected at the germination stage and 46 loci were identified for several morphological and physiological parameters at the seedling stage. Among them, nine and 33 loci with the ACC9 alleles increased salt tolerance at the germination and seedling stages, respectively. Moreover, several major QTLs were found to be co-localized in the same or overlapping regions of previously reported genes for salt stress. These major loci will facilitate improving salt-tolerance rice in genome-breeding programs.

## 1. Introduction

The world population is estimated to increase by more than two billion in the next 30 years while agricultural areas are facing a real decrease [1]. Agriculture production is principally influenced by prevailing unfavorable environmental conditions, such as drought and salinity, which are challenging global food security. Drought and salinity are the most serious factors that affect rice production worldwide [2]. It is estimated that more than 7% of arable land areas for crop cultivation are affected by salinity due to improper irrigation and fertilizer abuse [3]. It is an emergent need to develop tolerance varieties to combat the stresses and to sustain food production. Rice is one of the staple foods for two-thirds of the world population [4]. Hence, the increase in food production, particularly rice production, is urgent to meet the demand of the growing population. To increase rice production of saline-affected areas, identification of quantitative trait loci (QTLs) associated with salt tolerance is an essential step for the improvement in varieties of salt-tolerant rice.

Salinity is defined as the accumulation of a high concentration of salt, especially NaCl as well as other soluble salt ions in either soil or water, which has a deleterious effect on plant development and growth [5]. Rice is extremely susceptible to salt stress at the germination and seedling stages [6]. Salt tolerance of rice is a quantitative character controlled by many genes and influenced by environmental factors [7]. Significant efforts and considerable advancements have been made in the detection of QTLs to understand the genetic basis of salinity tolerance of rice [7,8,9,10,11,12]. Several QTLs associated with salinity tolerance have been identified at the germination and seedling stages in various genetic populations [10,11,12]. More than 20 QTLs related to salt sensitivity, sodium (Na^+^) concentration, and sodium-to-potassium (K^+^) ratio within roots and shoots are recorded on the GRAMENE website (https://archive.gramene.org). The locus *Saltol* was first detected on chromosome 1 and explained 43% of the total phenotypic variation in the Na^+^/K^+^ ratio of the seedling in the IR29/Pokkali recombinant inbred line population [13]. Sixteen QTLs for salt tolerance were detected by using an F_2_ population developed from Cheriviruppu × Pusa Basmati 1 at both the seedling and reproductive stages [11]. Recently, genome-wide association studies (GWAS) in natural populations of Asian cultivated rice (*Oryza sativa* L.) have been conducted to map QTLs for salt tolerance [14,15,16,17,18,19]. Moreover, some genes associated with salt tolerance were cloned in rice. For example, the *SKC1* (also known as *OsHKT1;5*) gene that encodes a high affinity K^+^ type transporter regulating K^+^/Na^+^ homeostasis has been cloned using a map-based approach [20]. The cation-chloride cotransporter gene (*OsCCC1*) associated with salt tolerance was identified with the role of maintaining K^+^ and Cl^−^ homeostasis in rice [21].

Africa rice (*Oryza glaberrima* Steud) carries many genes of desirable characters that can be transferred to Asian cultivated varieties, especially those responsible for resistance to biotic and abiotic stresses [22]. However, there are a limited number of studies using Africa rice to identify salt-associated QTLs. For example, Meyer et al. (2016) performed GWAS in a panel of germplasms that comprised 93 African rice varieties and found 11 regions significantly associated with six salt-tolerance traits at the seedlings stage, highlighting the value of this unique genetic resource [23]. To explore genetic variation of African rice in salt tolerance, the main objective of this study is to use a genome-sequenced Africa rice IRGC96717 (ACC9) and a genome-sequenced *indica* variety Zhenshan 97 (ZS97) to discover QTLs for salinity tolerance of the germination and seedling stages for improving rice varieties in breeding programs.

## 2. Results

### 2.1. Responses of ACC9 and ZS97 to Salt Stress

**Figure 1:**
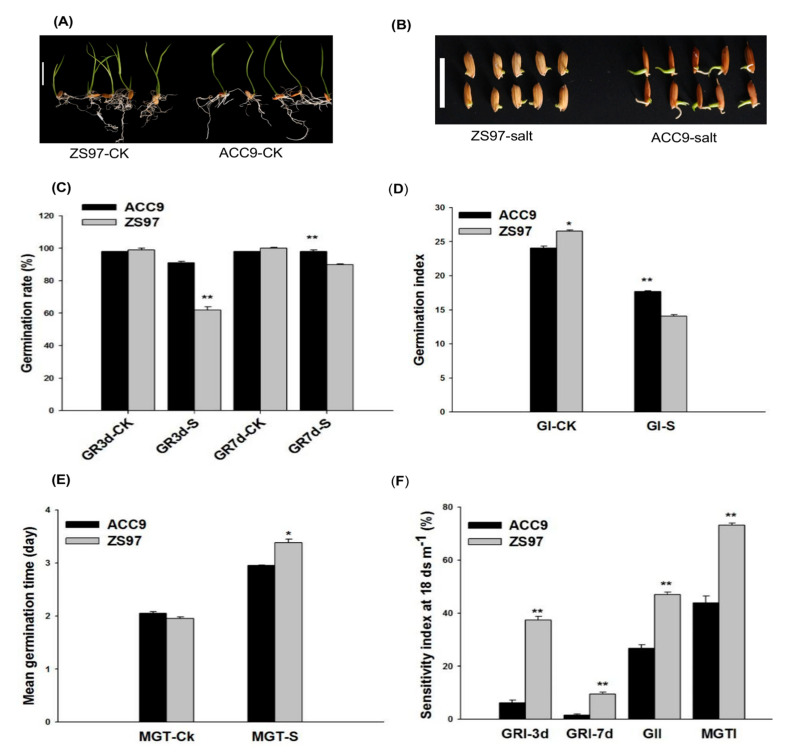
Seed germination parameters of the parental lines, ACC9 and ZS97, under control (CK) and salt treatment (S). Morphological differences of seed germination between ACC9 and ZS97 under control CK (**A**) and salt stress (**B**); (**C**) seed germination percentage at 3d and 7d after imbibition, (**D**) germination index (GI), (**E**) mean germination time (MGT), (**F**) sensitivity index. The error bars represent the means and standard errors of three replications. Asterisks (*, **) indicate significant differences according to Student’s *t*-test at *p* < 0.05 and 0.01, respectively. Scale bar = 2 cm in (**A**,**B**).

At the germination stage, the seed germination parameters (GR-3d, GR-7d) of ACC9 and ZS97 were similar under control conditions (CK) (Figure 1A); however, under salt stress (18 dS m^−1^ NaCl), ACC9 displayed better performance in terms of germination percentage than ZS97 (Figure 1B,C). ACC9 showed a significantly lower germination index (GI) than ZS97 under CK, but an opposite pattern was observed under salt stress (Figure 1D). ACC9 had a significantly lower mean of germination time (GT) than ZS97 under salt stress (Figure 1E). The relative values of the above four traits (GR-3d, GR-7d, GI, and GT) at the germination stage between salt stress and control were calculated and referred to as sensitivity index. The sensitivity indices revealed that ACC9 was more tolerant to salt stress than ZS97 at the germination stage (Figure 1F).

**Figure 2:**
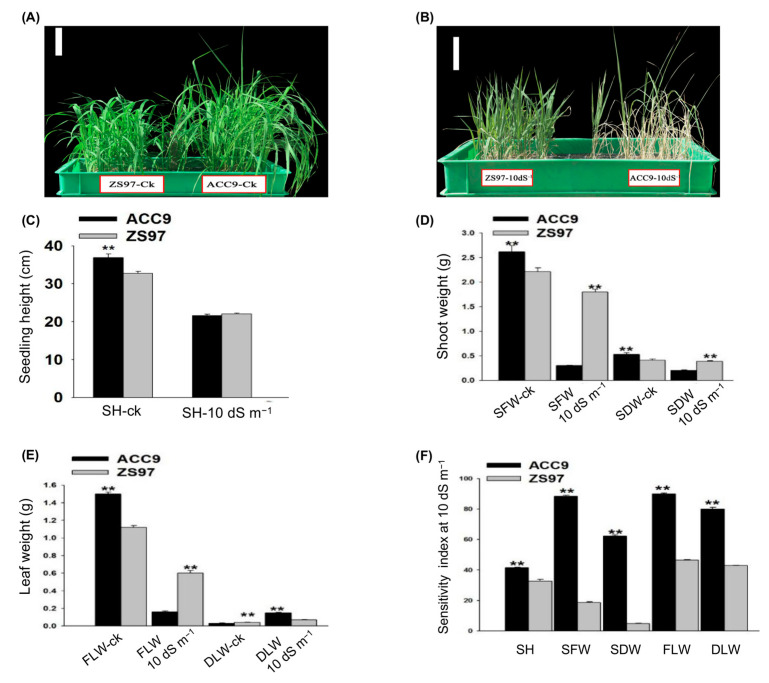
Seedling growth and biomass at 14 d after salt treatment with 10 dS m^−1^. Morphological differences of seedlings between ACC9 and ZS97 under control CK (**A**) and salt stress (**B**); (**C**) seedling height (SH); (**D**) shoot fresh (SFW) and dry weight (SDW); (**E**) fresh leaf weight (FLW) and dead leaf weight (DLW); (**F**), sensitivity index for SH, SFW, SDW, FLW, and DLW under stress. Values are the means and standard errors of two replications. Asterisks (**) represent significant differences according to Student’s *t*-test at *p* < 0.01, respectively. Scale bar = 10 cm in (**A**,**B**).

At the seedling stage, salt stress repressed plant growth of both parents (Figure 2). ACC9 displayed better performance in terms of seedling growth than ZS97 (Figure 2A), but the plant growth of ACC9 was more affected by the stress of 10 dS m^−1^ NaCl than ZS97 (Figure 2B). The differences between the two parents concerning seedling height (SH), shoot fresh weight (SFW), shoot dry weight (SDW), fresh leaf weight (FLW) and dead leaf weight (DLW) as well as their sensitivity indices are investigated. As shown in Figure 2 and Table S1, ACC9 had significantly higher SH, SFW, and SDW than ZS97 under control. There was no significant difference in SH between the two parents under salt stress (Figure 2C). Conversely, ZS97 had significantly higher SFW and SDW than ACC9 under salt stress (Figure 2D). ACC9 revealed significantly higher FLW than ZS97 under salt-stress conditions, while no significant DLW was observed under control conditions (Figure 2E). On the other hand, ZS97 had significantly higher FLW and lower DLW than ACC9 under stress conditions (Figure 2E). The sensitivity indices of the above five seedling traits in ACC9 were significantly higher than those in ZS97 (Figure 2F), suggesting that ACC9 may be less tolerant to salt stress than ZS97 in terms of seedling growth.

Concerning the biochemical traits, ACC9 had higher Na^+^ content and Na^+^/K^+^ ratio (NaKR) under control conditions but lower K^+^ content than ZS97, while ACC9 still had higher Na^+^ content and NaKR, but lower contents of K^+^ under salt stress than ZS97 (Table S1).

### 2.2. Phenotype Variation in BIL Population under Salt Stress

The backcross inbred line (BIL) population exhibited wide variations in germination parameters (GR-3d, GR-7d, GI, GT) and its sensitivity indices, which showed nearly normal distributions (Figure S1). At the seedling stage, SH, SFW, SDW, DLW, Na^+^ content, and K^+^ content also showed normal distributions (Figure S2). This indicates the assayed traits as quantitative traits are governed by multiple genes. In addition, the frequency distribution of the SH sensitivity index (SHI) was continuous but with 2 peaks.

Significant correlations were observed among many assayed traits at the germination and seedling stages: positive correlations among GR-3d, GR-7d, and GI under 18 dS m^−1^ NaCl; negative correlation between GT and GR-3d, and between GT and GI (Figure S3). In addition, there were positive and significant correlations between SH, SFW, and SDW under salt stress (Figure S3). SFW was negatively correlated with DLW and DLSFR under salt stress. Concerning the correlation between physiological and morphological traits, Na^+^ content and NaKR had significantly positive correlations with DLW and DLSFR, but revealed significantly negative correlations with SFW (Figure S3). Notably, the seedling traits were not significantly correlated with the germination traits under salt stress.

### 2.3. QTLs Identified for Germination Traits

A total of 23 QTLs were identified for four germination traits, which distributed on 9 chromosomes (Table 1). For GR-3d, three QTLs—*qGR-3d2*, *qGR-3d7*, and *qGR-3d12*—were identified under control conditions, and *qGR-3d12* explained the highest phenotypic variation (PVE) (9.5%) of germination percentage at 3d after seed imbibition (Table 1). For GR-7d, seven QTLs were identified, of which six loci had ACC9 alleles increasing germination percentage. Under salt stress, two QTLs were detected for the germination parameters; of them, *qGR-3d12* explained the largest phenotypic variation (24.5%).

Regarding germination index (GI), no QTL was identified under control conditions. One QTL, *qGI11*, was identified under salt stress (Table 1), explaining 11.5% of PVE. The additive effect of ZS97 allele increased GI. For GT, two QTLs, *qGT1.1* and *qGT1.2*, were detected under control, and two QTLs, *qGT4* and *qGT9,* were identified under salt stress (Table 1). The QTL *qGT1.1* explained 18.8% of PVE under control conditions, followed by *qGT4* (15.5%) under salt stress. The additive effect of the ACC9 alleles decreased GT at both loci. For the sensitivity index, three QTLs, *qGRI-3d12*, *qGRI-7d10*, and *qGTI4,* were identified, with the decreased effects from the ACC9 alleles at *qGRI-3d12* and *qMGTI4* (Table 1).

### 2.4. QTLs Identified for Morphological Traits at the Seedling Stages

QTLs detected for SH, SFW, SDW, DLW, DLSFR, and their sensitivity indices are shown in Table 2. One QTL, *qSH1,* was identified as a major locus for seedling height under both control and salt-stress conditions, explaining 61.9% of PVE under control and 57.1% of PVE under salt-stress conditions. Concerning the sensitivity index, two QTLs, *qSHI1* and *qSHI7,* were mapped on chromosome 1 and 7, respectively. The additive effect of the ACC9 alleles increased seedling height under control and stress conditions, and decreased its sensitivity index (Table 2).

One QTL, *qSDW1*, was detected for SDW and explained 28.6% of PVE under salt stress (Table 2). Three QTLs, *qDLW1*, *qDLW8.1* and *qDLW8.2*, were detected for DLW under control conditions. Moreover, three QTLs, *qDLW2*, *qDLW6* and *qDLW9,* were identified for DLW under salt stress. Of these, the alleles from ACC9 at all loci increased dead leaf weight. Seven QTLs, *qDLWI2*, *qDLWI4.1*, *qDLWI4.2*, *qDLWI6.1*, *qDLWI6.2*, *qDLWI9*, and *qDLWI10*, were detected for DLW sensitivity index (Table 2). The ACC9 alleles reduced dead leaf weight index at all detected QTLs.

Concerning QTLs for Na^+^ content, one QTL, *qNa1*, was mapped on chromosome 1 under control (Table 2). Under salt stress, two QTLs, *qNa2.1* and *qNa2.2*, were detected on chromosome 2, respectively with 5.3% and 17.8% of PVE. The additive effect of the ACC9 alleles decreased Na^+^ contents under both control and stress conditions. For K^+^ content, *qK1* and *qK10* were identified under control conditions, contributing 10.3% and 13.1% of PVE, respectively. Under salt stress, three QTLs, *qK2*, *qK6* and *qK11*, were detected with a range of 9.3% to 26.1% of PVE. The ACC9 alleles increased the K^+^ concentration of shoots. For Na^+^/K^+^ ratio, two QTLs (*qNaKR1* and *qNaKR11*) under control conditions and two QTLs (*qNaKR2.1* and *qNaKR2.2*) were detected under salt stress. The largest phenotypic variance (PVE = 19.1%) was explained by *qNaKR11* under control, followed by *qNaKR2.2* (17.8%) under salt stress conditions. The ACC9 alleles at these four detected loci decreased the Na^+^/K^+^ ratio.

### 2.5. Common QTLs Identified under Salt Stress

**Figure 3:**
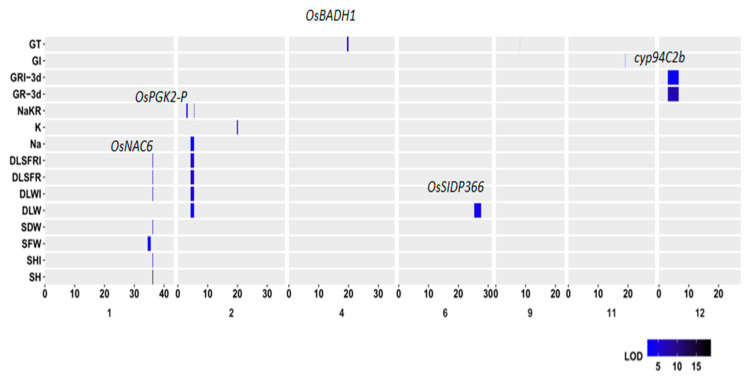
Locations of the common QTLs identified for germination and seedling traits in the backcross inbred line (BIL) population derived from ACC9 and ZS97. Some reported genes for salt tolerance are indicated. The trait abbreviations are explained in Table 2.

Several QTLs were identified with PVE ≥10% for the assayed traits under salt stress and colocated in the same or overlapping regions (Figure 3). For example, six QTLs, such as *qSH1*, *qSDW1*, and *qSHI1*, were located in a small bin (B01C254, 190 kb) on chromosome 1. Five QTLs, *qDLW2*, *qDLSFR2*, *qNa^+^2.2*, *qDLWI2*, and *qDLSFRI*2, were overlapped on the bin B02C61 in chromosome 2. Two common QTLs, *qGR-3d12* and *qGRI-3d12*, were located on B12C6 on chromosome 12. In addition, three QTLs—each with minor effect—*qDLSFR4.1*, *qDLWI4.1*, and *qDLSFRI4.1,* were colocalized in B04C175, whose size is 300 kb; and four QTLs, *qGR-7d4.5*, *qDLWI4.2*, *qDLSFR4.2,* and *qDLSFRI4.2,* were mapped on the same bin B04C302 (400 kb) (Table 1 and Table 2). There are two common QTL clusters located on chromosome 6. One cluster includes three QTLs, *qGR-7d6*, *qDLSFR6.1,* and *qDLWI6.1;* another cluster includes four QTLs, *qDLW6*, *qDLSFR6.2*, *qDLWI6.2,* and *qDLSFRI6*. Four QTLs, *qGR-7d10*, *qGRI-7d10*, *qDLSFR10,* and *qDLWI10* were overlapped on B10C43 on chromosome 10 (Table 1 and Table 2). These QTLs may be the genetic bases of significant correlations between GR, GI and GT at the germination stage, and among SH, SFW, SDW, DLW, DLSFR, Na^+^ content, K^+^ content and Na^+^/K^+^ ratio at the seedling stage.

## 3. Discussion

Previous studies have reported that the sensitivity of rice to salinity varies depending on different growth stages. Salt stress hinders germination rate, leading to inferior plant growth and reduced yield production [24]. Therefore, salinity tolerance of rice at the germination and seedling stages is an extremely important parameter in the improvement of rice productivity under salt stress, particularly in the regions where the cultivation mainly depends on direct-seeding systems [25]. Developing rice varieties of salinity tolerance at early growth stages is the utmost important step in rice breeding programs.

Our results showed that germination parameters were extremely affected by salt stress (18 dS m^−1^ NaCl) (Figure 1). They are consistent with previous reports [26]. Importantly, ACC9 was more tolerant to salt stress than ZS97 at the germination stage. The present results are in agreement with a previous study that salinity reduced the germination rate of ZS97. The reduction of germination rate was found to be due to a decrease in α-Amylase gene activity, which affects the bioactive GA in the germinating seeds [27]. Five major QTLs were identified, each explaining 10% and more of PVE in the germination characters in the BIL population. On the other hand, ZS97 showed salinity tolerance compared with ACC9 at the seedling stage (Figure 2). Eight major QTLs were identified for seedling growth and biomass. It is unexpected that there are no significant correlations between the germination parameters and the seedling biomass under salt-stress conditions (Figure S3). In line with this, the common loci for the germination and seedling traits were hardly identified with the BIL population under the stress conditions (Figure 3). This insignificant correlation between the germination parameters and seedling biomass under salt stress is consistent with the previous study [18]. Therefore, ZS97 would be used as an elite tolerance germplasm for the improvement in rice salt tolerance at the seedling stage and ACC9 as a tolerance source for germination ability.

Mapping of QTLs associated with salt tolerance is considered the basic step in marker-assisted breeding of salt-tolerance varieties. In the current study, the BIL population derived from the Africa rice ACC9 and *indica* cultivar ZS97 was used to identify the QTLs associated with salt tolerance at early growth stages. Among 23 QTLs identified at the germination stage, nine loci with ACC9 alleles increased salt tolerance; among 46 QTLs detected at the seedling stage, 33 loci with ACC9 alleles enhanced salt tolerance. Africa rice ACC9 was reported to be a suitable donor for many desirable traits such as drought tolerance and early vigor characteristics [28,29]. Moreover, several major QTLs (with PVE ≥ 10%) for different traits were found to be colocalized in the same or overlapping regions (Figure 3), indicating there is the same gene or linked genes having a pleiotropic effect on these multiple traits. For example, three loci (*qSH1*, *qSDW1* and *qSHI1*) colocalized in B01C254; five loci (*qDLW2*, *qNa^+^2.2*, *qDLSFR2*, *qDLWI2*, and *qDLSFRI2*) overlapped in B02C61. These colocalized QTLs for various traits could be very beneficial for a rice breeding program via marker-assisted selection to improve salt tolerance.

Some loci in the current study were identified in small bins (or regions) in the BIL population and localized within or near previously reported genes or loci associated with salt tolerance in rice (Figure 3; Table 1 and Table 2), which allowed naming of candidate genes at the given bin using the available gene annotation database (http://rice.plantbiology.msu.edu/). For example, six major loci colocalized in B01C254 within a 190-kb size encompassing two known genes: the green revolution gene *sd1* [30] and *SNAC6* [31]. As *qSH1* explained the highest phenotypic variation in seedling height under salt stresses, the gene *sd1* may be the most likely candidate for *qSH1*. *qNaKR2.1* was found near *OsPgk2* (Figure 3), which was extremely induced by salt stress in a salt-tolerance variety Pokkali [32]; *qGT4* was found in proximity to *OsBADH1*, which could enhance rice adaptability to salt stress [33]. In addition, *qDLW6* for dead leaf weight was located within the same region of previously identified *qDSW6.2* [34]. The *qDLW6* region harbors a candidate gene *OsSIDP366*, which was reported to be a positive regulator responding to drought and salt stress in rice [35]. *qGR-3d12* and *GRI-3d12* are located in B12C6 (Figure 3), where *CYP94C2b* (LOC_Os12g05440) was reported to enhance salt tolerance of rice [36]. Thus it may be a possible candidate gene. Among these loci of interest, three loci (*qSHI1*, *qGT4* and *qGRI-3d12*) with salt tolerance alleles from ACC9 could be interpreted as promising ones for the improvement in salt tolerance of Asian cultivated rice. These data indicate that the identified QTLs with possible candidate genes could be useful for developing varieties of salinity tolerance at early seedling stages in rice breeding programs.

## 4. Materials and Methods

### 4.1. Plant Materials

The population used at the present study is a backcross inbred line (BIL) produced by single-seed descend method from a backcross between an Africa landrace IRGC96717 (ACC9) as donor and an *indica* cultivar Zhenshan 97 (ZS97) as recurrent parent. The first backcross (BC1F1) had been self-crossed for 11 generations (BC_1_F_11_) (Figure S4). The BIL population was grown at the experimental field of Huazhong Agricultural University in 2018 from May to September at Wuhan (30.48° N, 114.2° E), China. The seeds of each line were harvested at 35 days after heading and equilibrated in a storage chamber with low relative humidity (18% RH) for further analyses.

### 4.2. Germination Evaluation of the Parents and BIL Population

Germination tests under salt conditions were conducted using 50 healthy seeds per sample in a petri plate containing three layers of filter paper moistened with 15 mL of 18 dS m^−1^ NaCl solutions [26,37]. As a control, a germination test was carried out using 15 mL distilled water instead of the saline solution. Germination tests under both the saline and control conditions were kept in a growth chamber with 12 h light/12 h dark circle, 25 ± 2 °C, and 60% RH. The germinated seeds were recorded every 24 h for seven days. The germination experiment was repeated three times for each sample of the parents and BILs. Germination rate (GR), germination index (GI) and mean germination time (GT) were calculated as described previously [34].

### 4.3. Evaluation of Seedling Performance of the Parents and BIL Population

Thirty uniformly germinated seeds were transferred to planting plates filled with moist soil. The seedlings at 14d after seed sowing were then transplanted into hydroponics solution [38] and grown in a greenhouse, where room temperature ranged from 28 to 30 °C during the day and 18 to 23 °C during the night. Relative humidity was set at 85%, and light set at 671 µmol·m^−2^·S^−1^ with a minimum of 100 and a maximum 1400 µmol·m^−2^·s^−1^ during the day [25].

For evaluation of salt tolerance, 14 day-old seedlings were treated two weeks with salinized nutrient solution with the level of NaCl (10 dS m^−1^) for BIL lines. The nutrient solutions were replenished every 3d. The pH range was fixed from 5.00 to 5.50. Seedling performances were recorded after 14d of NaCl application. Five traits, seedling height (SH), shoot fresh weight (SFW), shoot dry weight (SDW), dead leaf weight (DLW) and dead leaf to shoot fresh weight ratio (DLSFR), were determined as described previously [34]. All collected samples were dried immediately at 70 °C for 72 h using a forced-air oven [25]. The relative values of the aforementioned traits (the sensitivity index) assayed at the seedling stage between the salt stress and control were calculated according to the formula [39] (Equation (1)):

(1) SI=(control value−treatment)/ control value ×100

Na^+^ and K^+^ contents in dry shoot samples were measured according to the method reported previously [40]. Approximately 1 g of each sample was put into a 50 mL centrifuge tube with 20 mL of 1N HCL for digestion and kept the sample in a water bath at 85 °C for 30 min with shaking manually each 10 m interval. The homogenized samples were filtered using Whatman papers and kept at room temperature overnight. Na^+^ and K^+^ contents were measured using a FP6431 flame photometer (Shanghai Instrument, China) as reported previously [40].

### 4.4. Linkage Map Construction and QTL Analysis

The CTAB method was applied to extract DNA from young seedling leaves [41]. A genotyping-by-sequencing strategy was used to analyze the genotypes of BIL population with a minor modification [42]. Briefly, two restriction enzymes, *EcoR*I and *Msp*I, were used to digest the genome DNA into fragments. Single-nucleotide polymorphism (SNP) calling and real site frequency spectrum (realSFS) were performed based on estimation of site frequency. A Practical Extraction and Report Language (PERL) was used for filtration SNPs as well as indels taking into consideration of the following criteria: the missing data greater than 50% and allele frequency less than 5% in the population. According to SNP genotyping, 714 bin markers covered the rice genome were used for constructing molecular linkage map. Bin name and order on different chromosomes of ACC9/ZS97 population are presented in the Supplemental Table S2. IciMapping software with inclusive composite interval mapping method was used for QTL analysis [43]. The existence of a QTL with an additive effect was declared by the logarithm of odds (LOD) threshold of 2.5. Correlations of traits, mean and standard error were implemented using the software SPSS version 23 (SPSS Inc., Chicago, IL, USA).

## 5. Conclusions

Salinity is one of the most important abiotic stresses that influence the global production of rice. Salt tolerance is a quantitative character that depends upon the cumulative action of many genes. In this study, germination parameters and seedling growth were used to evaluate salt tolerance of the BILs derived from the Africa rice ACC9 and *indica* cultivar ZS97. A total of 23 and 46 QTLs were identified for the assayed traits associated with salt tolerance at the germination and seedling stages, respectively. At the germination stage, three major QTLs, *qGT4*, *qGR-3d12,* and *qGRI-3d12,* explained more than 15% of the phenotypic variance on corresponding traits with ACC9 alleles enhancing germination parameters under salt stress. At the seedling stage, major common QTL regions (e.g., *qSH1*, *qSDW1*, and *qSHI1)* were identified for multiple traits, with the ACC9 alleles showing increased seedling growth under salt stress. In addition, at least three loci were identified in small bins (or regions) and localized in or nearby previously reported genes associated with salt stress. These data of the promising loci with a possible candidate gene will be useful for improving rice with salt tolerance through the genomic breeding approaches.

## Figures and Tables

**Table 1:** Putative QTLs identified for salinity tolerance in the BIL population at the germination stage.

Treat	Traits ^a^	QTL	Chr	Bin ^b^	Left Marker (Mb)	Right Marker (Mb)	Bin Size (Mb)	Add ^c^	LOD ^d^	PVE (%) ^e^
Control	GR-3d	*qGR-3d2*	2	B02C46	4.26	5.43	1.17	−0.13	5.6	6.4
		*qGR-3d7*	7	B07C21	16.27	22.84	6.56	−0.05	2.7	1.9
		*qGR-3d12*	12	B12C7	2.98	6.62	3.65	−0.14	12.9	9.5
	GR-7d	*qGR-7d4.1*	4	B04C67	10.72	11.07	0.34	−0.09	3.6	5.8
		*qGR-7d4.2*	4	B04C134	16.33	16.41	0.08	−0.10	4.1	5.6
		*qGR-7d4.3*	4	B04C254	17.14	17.17	0.03	−0.09	4.3	5.7
		*qGR-7d4.4*	4	B04C348	18.73	18.83	0.10	−0.07	9.1	5.1
		*qGR-7d10*	10	B10C43	0.61	13.89	13.28	−0.10	7.3	5.8
		*qGR-7d11*	11	B11C9	2.29	7.39	5.10	−0.09	2.8	5.2
		*qGR-7d12*	12	B12C45	19.54	20.38	0.83	0.05	5.2	2.6
	GT	*qGT1.1*	1	B01C105	11.93	11.96	0.03	0.21	5.4	18.8
		*qGT1.2*	1	B01C236	33.47	33.59	0.12	0.21	3.0	13.1
Stress	GR-3d	*qGR-3d4*	4	B04C372	24.68	27.20	2.51	0.09	2.7	6.0
		*qGR-3d12*	12	B12C6	2.98	6.62	3.65	0.20	7.3	24.5
	GR-7d	*qGR-7d4.5*	4	B04C302	18.27	18.67	0.40	−0.22	3.3	5.1
		*qGR-7d6*	6	B06C46	4.62	5.20	0.58	−0.25	7.5	5.1
		*qGR-7d10*	10	B10C43	0.61	13.89	13.28	−0.22	3.3	5.1
	GI	*qGI11*	11	B11C55	19.02	19.10	0.08	−1.95	2.6	11.5
	GT	*qGT4*	4	B04C353	19.46	19.97	0.51	−0.34	6.1	15.5
		*qGT9*	9	B09C180	8.11	8.13	0.02	−0.24	4.1	10.8
Index	GRI-3d	*qGRI-3d12*	12	B12C6	2.98	6.62	3.65	−12.28	3.3	11.5
	GRI-7d	*qGRI-7d10*	10	B10C43	0.61	13.89	13.28	7.09	3.8	4.9
	GTI	*qGTI4*	4	B04C183	16.41	16.72	0.31	−19.00	3.3	9.0

Putative QTLs at germination stage: ^a^, GR-3d and GR-7d, germinated seed percentage at 3rd and 7th day after imbibition, respectively; GI germination index; GT mean germination time; GRI-3d, GRI-7d, GII and GTI represent the sensitivity index of GR-3d, GR-7d, GI, and GT, respectively; ^b^, location of quantitative trait loci (QTLs) on chromosome; ^c^, additive effect of the ACC9 allele; the negative value indicating the ACC9 allele decreased the trait; ^d^, logarithm of odds; and ^e^, the phenotypic variance explained by each QTL.

**Table 2:** Putative QTLs identified for salinity tolerance in the BIL population at the seedling stage.

Treats	Traits ^a^	QTL	Chr	Bin ^b^	Left Marker	Right Marker	Bin Size	Add ^c^	LOD ^d^	PVE (%) ^e^
Control	SH	*qSH1*	1	B01C254	36.09	36.28	0.19	7.78	21.4	61.9
	SFW	*qSFW1.1*	1	B01C254	36.09	36.28	0.19	0.17	11.7	34.8
		*qSFW4*	4	B04C322	18.73	8.83	0.10	0.09	3.0	7.2
	DLW	*qDLW1*	1	B01C254	36.09	36.28	0.19	0.01	6.5	11.5
		*qDLW8.1*	8	B08C30	1.94	2.20	0.26	−0.01	8.8	16.4
		*qDLW8.2*	8	B08C40	6.17	6.54	0.37	0.01	4.2	7.0
	Na^+^	*qNa 1*	1	B01C273	38.50	38.71	0.21	0.40	3.1	3.6
	K^+^	*qK1*	1	B01C73	7.73	8.26	0.53	1.34	3.1	10.3
		*qK10*	10	B10C43	0.61	13.89	13.28	1.61	3.8	13.1
	Na^+^/K^+^	*qNaKR1*	1	B01C269	37.81	37.95	0.13	−0.02	3.1	9.8
		*qNaKR11*	11	B11C92	25.75	25.86	0.11	−0.02	5.8	19.1
Stress	SH	*qSH1*	1	B0C254	36.09	36.28	0.19	5.08	18.4	57.1
	SFW	*qSFW1.2*	1	B01C236	34.49	35.50	1.01	0.06	3.2	15.2
	SDW	*qSDW1*	1	B01C254	36.09	36.28	0.19	0.02	7.3	28.6
	DLW	*qDLW2*	2	B02C61	4.26	5.43	1.17	0.02	3.5	10.0
		*qDLW6*	6	B06C161	25.32	27.64	2.32	0.02	4.4	10.2
		*qDLW9*	9	B09C28	0.66	0.68	0.01	0.01	2.6	2.0
	DLSFR	*qDLSFR2*	2	B02C61	4.26	5.43	1.17	0.08	6.3	4.3
		*qDLSFR4.1*	4	B04C175	16.41	16.72	0.31	0.09	3.3	4.1
		*qDLSFR4.2*	4	B04C302	18.27	18.67	0.40	0.09	4.0	4.0
		*qDLSFR6.1*	6	B06C49	4.62	5.20	0.58	0.09	3.5	3.3
		*qDLSFR6.2*	6	B06C138	25.32	27.64	2.32	0.09	5.7	4.2
		*qDLSFR9*	9	B09C90	2.40	2.53	0.13	0.03	2.7	0.8
		*qDLSFR10*	10	B10C43	0.61	13.89	13.28	0.09	5.0	4.2
	Na^+^	*qNa2.1*	2	B02C22	4.15	4.26	0.11	−4.37	3.2	5.3
		*qNa2.2*	2	B02C61	4.26	5.43	1.17	−6.80	3.2	17.8
	K^+^	*qK2*	2	B02C180	19.80	20.15	0.35	1.95	8.0	26.1
		*qK6*	6	B06C0	0.11	0.15	0.04	1.15	3.4	9.3
		*qK11*	11	B11C92	25.75	2.59	0.11	1.23	3.4	9.6
	Na^+^/K^+^	*qNaKR2.1*	2	B02C20	2.82	3.27	0.44	−0.23	4.2	10.4
		*qNaKR2.2*	2	B02C110	5.43	5.56	0.13	−0.23	6.4	17.4
Index	SHI	*qSHI1*	1	B01C254	36.09	36.28	0.19	−3.42	6.1	16.4
		*qSHI7*	7	B07C3	2.48	8.21	5.73	−3.10	3.0	8.0
	DLWI	*qDLWI2*	2	B02C61	4.26	5.43	1.17	−0.94	5.4	2.8
		*qDLWI4.1*	4	B04C175	16.41	16.72	0.31	−0.94	4.9	2.7
		*qDLWI4.2*	4	B04C302	18.27	18.67	0.40	−0.96	6.7	4.0
		*qDLWI6.1*	6	B06C48	4.62	5.20	0.58	−0.99	2.8	2.6
		*qDLWI6.2*	6	B06C143	25.32	27.64	2.32	−0.95	5.1	2.7
		*qDLWI9*	9	B09C105	3.25	3.30	0.05	−0.37	3.2	0.6
		*qDLWI10*	10	B10C43	0.61	13.89	13.28	−0.95	5.4	2.8
	DLSFRI	*qDLSFRI2*	2	B02C61	4.26	5.43	1.17	−2.44	6.0	5.3
		*qDLSFRI4.1*	4	B04C175	16.41	16.72	0.31	−2.44	3.9	5.2
		*qDLSFRI4.2*	4	B04C302	18.27	18.67	0.40	−2.48	7.3	5.1
		*qDLSFRI6*	6	B06C140	25.32	27.64	2.32	−2.48	5.1	5.3
		*qDLSFRI9*	9	B09C100	2.71	2.97	0.26	−1.03	3.2	1.2
		*qDLSFRI10*	10	B10C43	0.61	13.89	13.28	−2.47	4.3	5.2

Putative QTLs at seedling stage: ^a^, SH, seedling height; SFW, shoot fresh weight; SDW, shoot dry weight; DLW, dead leaf weight; DLSFR, dead leaf to shoot fresh weight ratio; Na^+^, Na^+^ content in shoot; K^+^, K^+^ content in shoot; SHI, DLWI, and DLSFRI represent the sensitivity index of SH, DLW, and DLSFR, respectively; ^b^, location of QTLs on chromosome; ^c^, additive effect of the ACC9 allele; ^d^, logarithm of odds; and ^e^, the phenotypic variance explained by each QTL.

## Data Availability

Data is contained within the article and supplementary material.

## Supplementary Materials

The following are available online at https://www.mdpi.com/2223-7747/10/3/428/s1, Figure S1: Frequency distribution of germination parameters in the BIL population derived from ACC9 x ZS97 under salt stress with 18 dS m^−1^ NaCl. Figure S2: Frequency distribution of seedling traits in the BIL population derived from ACC9 x ZS97 under 10 dS m^−1^ NaCl salt stress. Figure S3: Correlation of morpho–physiological traits examined in the BIL population derived from ACC9 x ZS97 under salt stress at the germination and seedling stages. Figure S4: Construction procedure of the ACC9/ZS97 BIL population. Table S1: Morphological and physiological traits of ACC9 and ZS97 and BILs under control and salt stress conditions. Table S2: Bin information of ACC9/ZS97 BIL population.
