# Supplementary material for: Identification of QTLs for Salt Tolerance at the Germination and Seedling Stages in Rice
Source: Plants (Basel). 2021 Feb 24;10(3):428. doi: 10.3390/plants10030428 (PMC7996262; doi:10.3390/plants10030428)
Supplement: Supplementary file 1 [file plants-10-00428-s001.zip › Supplemental fig S1-3 and table S1.pdf]

Identification of QTLs for salt tolerance at the germination and seedling stages in rice

Walid R Nakhla[1, 2], Wenqiang Sun[1, 2], Kai Fan [1, 2], Kang Yang[1, 2] , Chaopu Zhang [1,2] and Sibin Yu[1 2,*]

[1] National Key Laboratory of Crop Genetic Improvement, Huazhong Agricultural University, Wuhan 430070, China

[2] College of Plant Science and Technology, Huazhong Agricultural University, Wuhan 430070, China

* Correspondence: Sibin Yu (ysb@mail.hzau.edu.cn)

Supplemental Figures S1-S3, Table S1

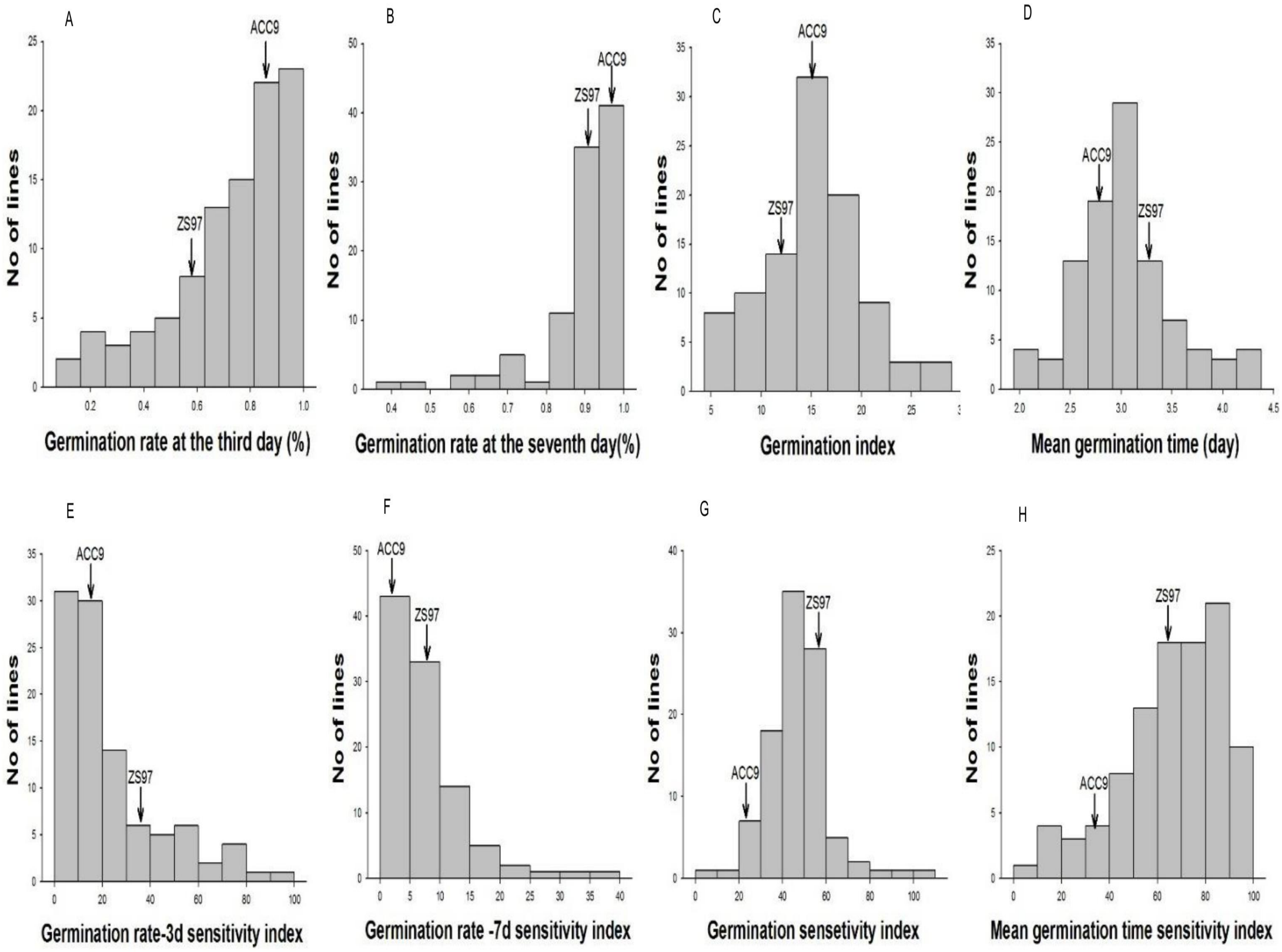

Figure S1. Frequency distribution of germination traits in the BIL population derived from ACC9 x ZS97 at the germination stage under 18dS m^{-1} NaCl salt stress. Parental means are indicated by arrows. (A, B) Germination percentage at 3rd and 7th day after imbibition, respectively; (C) Germination index; (D) Mean germination time; (E-H) The sensitivity index of the above traits, respectively.

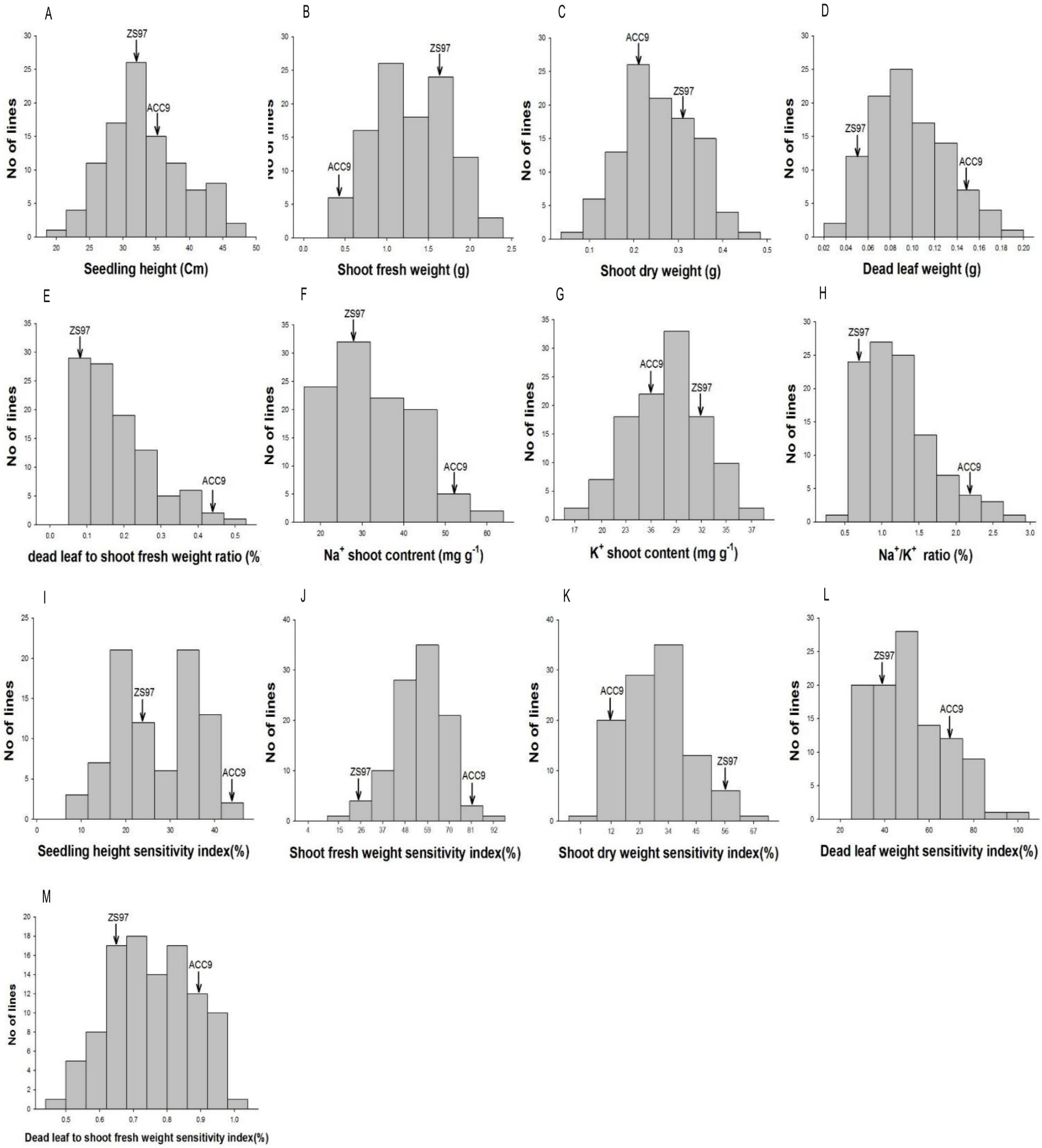

Figure S2. Frequency distribution of seedling traits in the BIL population derived from ACC9 x ZS97 at the seedling stage under 10dS m-1 NaCl salt stress. Means of the parental lines are indicated by arrow.

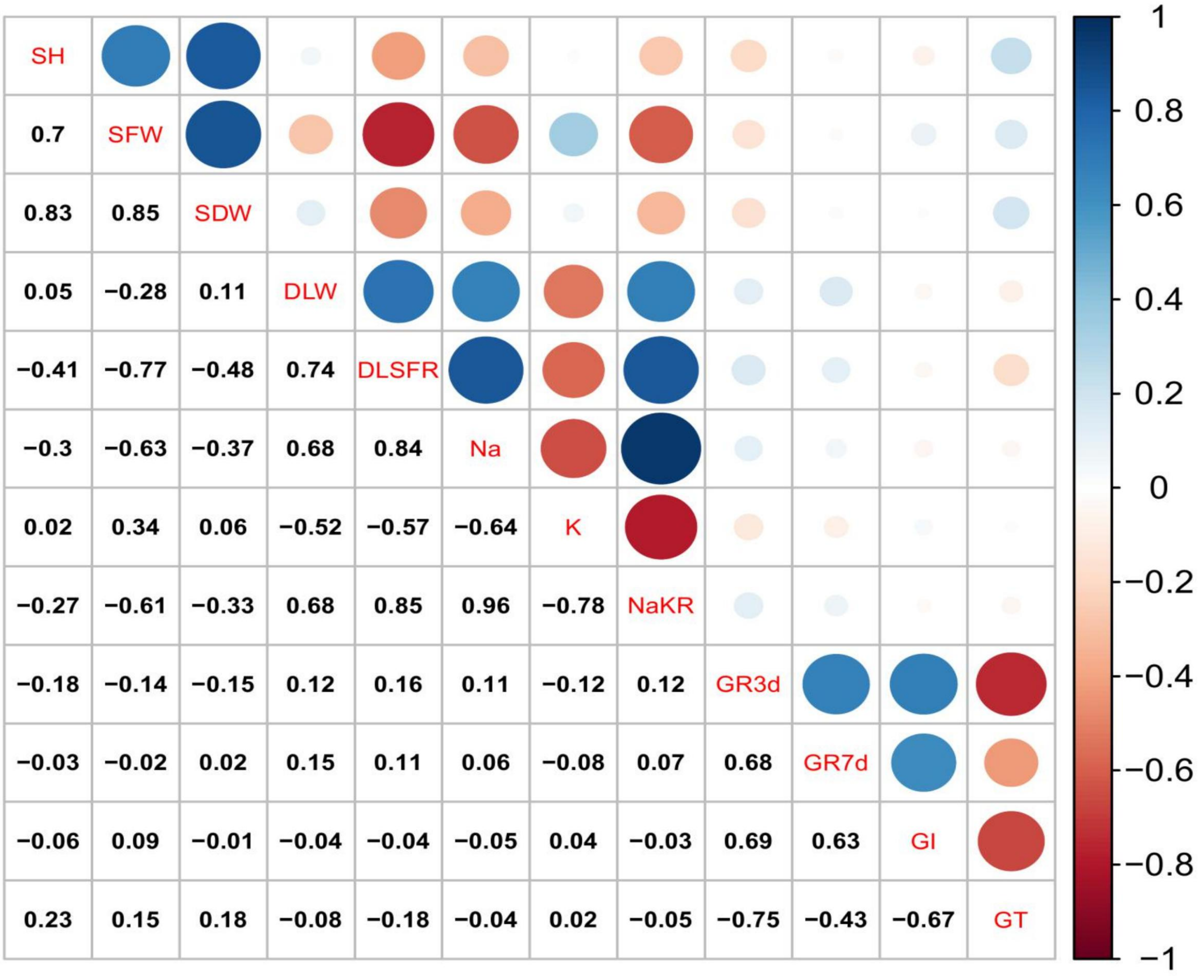

Figure S3. Correlation of morpho-physiological traits examined in the BIL population derived from ACC9 x ZS97 under salt stress at the germination and seedling stages. SH seedling height, SFW shoot fresh weight, SDW shoot dry weigh, DLW dead leaf weight, DLSFR dead leaf to shoot fresh weight ratio, Na content of Na^{+} in shoot, Kcontent of K^{+} in shoot; NaKR Na+/K+ ratio. GR3d, GR7d represent germination rate at 3d and at 7d after imbibition, respectively; GI germination index; GT mean germination time.

Table S1 Morphological and physiological traits of ACC9 and ZS97 and BILs under control and salt stress conditions

**Stages**	**Treatments**	**Traits**	**Parents**		**BILs**		
Germination			ACC9	ZS97	Min	Max	Mean
	CK	GR-3d (%)	98.0±1.0	99.0±0. 1	49.0	100.0	92.0
		GR-7d (%)	99.0±1.0	100.0±0.05	58.0	100.0	94.0
		GI	24.08±0.25**	26.54±0.04	6.29	43.75	27.5
		GT	2.05±0.03	1.95±0.03	1.23	3.04	1.8
	18 dS m^{-1}	GR-3d (%)	91.0±1.0**	62.0±2.8	0.00	1.00	72.0
		GR-7d (%)	98.0±1.0**	90.0±0.03	0.00	1.00	88.0
		GI	17.66±0.16**	14.08±0.23	4.31	29.00	15.0
		GT	2.95±0.01*	3.38±0.07	1.96	4.38	3.0
		GRI-3d	7.14± 1.02**	37.37±1.36	0.00	91.09	22.7
		GRI-7d	1.5±0.52**	10.0± 0.91	0.00	36.15	7.2
		GII	26.66+1.42**	46.94±0.97	0.00	100.00	45.3
		GTI	43.90±2.63**	73.33±0.63	0.00	97.33	66.7
Seedling	CK	SH(cm)	37.35±0.26**	33.20±0.50	22.65	61.26	42.0
		SFW(g)	2.65±0.02**	2.23±0.02	0.41	2.73	1.0
		SDW(g)	0.55±0.01**	0.43±0.01	0.06	0.56	0.2
		DLW	0.038±0.01	0.030±0.004	0.01	0.07	0.1
		DLSFR	0.014±0.005	0.013±0.003	0.01	0.08	0.1
		Na^+	5.81±0.02**	5.04±0.01	3.84	8.76	5.7
		K^+	30.88±0.05	35.66±.68**	23.64	40.54	31.4
		Na^+/K^+	0.19±0.002**	0.14±0.004	0.11	0.30	0.2
	10 dS m^{-1}	SH(Cm)	22.10±0.23**	23.50±0.06	19.07	45.40	33.0
		SFW(g)	0.33±0.01**	1.85±0.02	0.17	1.86	0.5
		SDW(g)	0.19±0.01**	0.41±0.03	0.06	0.45	0.1
		DLW	0.071±0.01**	0.050±0.01	0.03	0.19	0.1
		DLSFR	0.22± 0.03**	0.027± 0.01	0.03	0.50	0.2
		Na^+	46.20±0.11**	20.63±0.22	12.58	59.18	28.6
		K^+	23.19±0.14**	32.05±0.15	17.75	36.84	27.2
		Na^+/K^+	1.99±0.01**	0.64±0.01	0.39	2.76	1.09
		SHI (%)	40.83±0.20**	29.18±1.25	0.04	44.0	20.8
		SFWI (%)	87.54±0.30**	17.04±0.13	4.70	88.7	49.9
		SDWI (%)	66.61±2.82*	5.81±0.06	1.09	66.7	20.9
		DLWI (%)	-0.86±0.04**	-0.66±0.01	-4.35	-0.05	-1.12
		DLSFRI (%)	93.45±0.20	50.17±0.77	47.0	97.0	72.0

GR-3d and GR-7d, germinated seed percentage at 3rd and 7th day after imbibition, respectively; GI, germination index; GT, mean germination time; GRI-3d, and GRI-7d represent the sensitivity index of GR-3d and GR-7d, respectively; GII, sensitivity index of GI; and GTI, sensitivity index of GT; SH, seedling height, SFW, shoot fresh weight; SDW, shoot dry weight; DLW dead leaf weight; DLSFR, dead leave to shoot fresh ratio; Na^+, Na^+ content in shoot; K^+, K^+ content in shoot; Na^+/K^+, Na^+ to K^+ ratio; SHI, SFWI, and SDWI represent the sensitivity index of SH, SFW, and SDW, respectively. CK, 18 dS m^{-1}, and 10dS m^{-1} represent the control condition and two levels of NaCl treatments, respectively. Asterisks (*, **) indicate significant differences according to Student's *t*-test at $p < 0.05$ and 0.01, respectively
